# Examining uncertainty in journal peer reviewers’ recommendations: a cross-sectional study

**DOI:** 10.1098/rsos.240612

**Published:** 2024-09-11

**Authors:** Adrian Barnett, Liz Allen, Adrian Aldcroft, Timothy L. Lash, Victoria McCreanor

**Affiliations:** ^1^ School of Public Health & Social Work, Queensland University of Technology, Brisbane, Australia; ^2^ Taylor & Francis, London, UK; ^3^ Policy Institute, King’s College London, London, UK; ^4^ BMJ Publishing Group, London, UK; ^5^ Department of Epidemiology, Rollins School of Public Health, Emory University, Atlanta, GA, USA; ^6^ School of Medicine and Public Health, University of Newcastle, Callaghan, Australia

**Keywords:** peer review, uncertainty, decision-making

## Abstract

The peer review process is used throughout science but has often been criticized for being inconsistent, with decisions dependent on the peers who did the reviewing. Much of the decision inconsistency arises from the differences between reviewers in terms of their expertise, training and experience. Another source of uncertainty is within reviewers as they must make a single recommendation (e.g. ‘Accept’), when they may have wavered between two (e.g. ‘Accept’ or ‘Reject’). We estimated the size of within-reviewer uncertainty using post-review surveys at three journals. We asked reviewers to think outside the recommendation they gave (e.g. ‘Accept’) and assign percentages to all other recommendations (e.g. ‘Major revision’). Reviewers who were certain could assign 100% to one recommendation. Twenty-three per cent of reviewers reported no uncertainty (95% confidence interval 19–27%). Women were associated with more uncertainty at one journal, and protocol papers were associated with more uncertainty at one journal. Reviewers commonly experience some uncertainty when peer-reviewing journal articles. This uncertainty is part of the variability in peer reviewers’ recommendation.

## 1. Background

Peer review is an integral part of science. It controls what papers appear in the most prestigious journals and strongly influences scientists’ careers. Early career researchers are acutely aware that their careers can be dependent on a single peer review decision [1]. Despite the importance of peer review, there are many gaps in our understanding, including how to determine high-quality peer review and how journal policies influence peer review [2].

In broad terms, the peer review process at journals is for 2 to 4 peers to review the paper and provide their recommendation or score and comments. These reviews are then distilled by an editor who makes a decision which is broadly accept, revise or reject. This commonly used system has been criticized for lacking evidence of its effectiveness [2,3], being slow [3,4], costly [3,4], conservative [5], unable to detect errors [6–8], lacking transparency [7,9], frequently unprofessional [10] and prone to bias [3,11–13].

Peer review has also been criticized for being inconsistent [3,14]. This inconsistency arises because there is a ‘luck of the draw’ in terms of which reviewers are selected and available. Every reviewer has their own biases and experiences that influence their reviews. An ideal peer review system would give reliable decisions that are similar when the process is repeated, although decisions will never be perfectly reliable because of the healthy diversity in scientists’ expertise and experience [15]. Empirical work on the reliability of peer review has so far only examined the variability *between* reviewers, often by examining the agreement of independent reviewers examining the same papers [5,16–22].

Another source of uncertainty is *within* reviewers. This uncertainty can occur because reviewers usually make a single recommendation, e.g. ‘Accept’, but they may have considered other recommendations, e.g. ‘Minor revision’, and their recommendation could be dependent on circumstance, for example, reviewing the paper on a different day [23] or when tired [24]. Reviewers can struggle to commit to a single recommendation, such as in these three quotes from peer reviewers:

— ‘I also find it hard to say “reject” even though I know in my heart that a project is fundamentally flawed’ [25].
— ‘I hope this review is not too harsh and if so please let me know and we can adjust’ [26].
— ‘I am going to say REJECT, but I can imagine a MAJOR REVISIONS’ (their capitals) [26].

There are many potential reasons for this uncertainty. Some reviewers may want to give their colleagues the benefit of the doubt, as indicated in the above quotes. A reviewer may be uncertain about parts of the paper if their expertise does not cover all the methods used. Reviewers may be unfamiliar with the journal’s guidelines for reviewers, which vary between journals and can be time-consuming to read [27].

Reviewers have reflected on their decision-making processes as being ‘intuitive’ and ‘intangible’, implying some uncertainty in their recommendations [28]. In this study, we aimed to empirically measure within-reviewer uncertainty and hence discuss variability in peer review.

Reviewer uncertainty could never be reduced to zero. Unlike bias in peer review, some level of uncertainty is acceptable and may reflect genuine unknowns or disagreements about the work [15]. However, if uncertainty is larger (on average) for some papers or reviewers, then this may highlight where improvements to reliability could be made. For example, if there are particular paper types with higher levels of uncertainty.

To our knowledge, this is the first study to examine uncertainty within reviewers by asking them to estimate their uncertainty. A suggestion has been made to ask peer reviewers to give their confidence in their own reviews but in the context of questionable research practices not uncertainty [29]. The peer review for some conferences has included reviewers’ confidence scores on a numeric scale [30].

Our study team combined health and medical researchers, journal editors, and journal staff. Journals have been encouraged to engage in research on peer review [31–33] and this research aims to be part of the reflection on peer review [34].

## 2. Methods

### 2.1. Data collection

We approached peer reviewers at three journals: *BMJ Open*, *Epidemiology* and *F1000Research* (see electronic supplementary material, S1, for some background information on the journals). After they submitted their review, all reviewers were asked to participate in a study about the peer review process. Interested reviewers who clicked on the link were shown the following question:

**Table:** 

We want to know how certain you were about your recommendation and if you considered other recommendations. Can you estimate your probability of selecting the four review recommendations? Please provide your answers as percentages.	
Accept	0
Minor revisions	0
Major revisions	0
Reject	0
Total	0

The question aims to give a numerical estimate of uncertainty via their subjective assessment [35]. A reviewer who feels no uncertainty can assign 100% to one of the recommendations. Any per cent over zero and less than 100% indicates some uncertainty, which can be expressed both by (i) how many other recommendations researchers assign some non-zero percentage, with more recommendations indicating more uncertainty and by (ii) the spread of the non-zero percentages, with a greater spread indicating greater uncertainty. Reviewers were given two examples to help them complete the question; see electronic supplementary material, S2, for the questions. The question above is for the four recommendations used at *BMJ Open*, and there were alternative versions for *Epidemiology* and *F1000Research* to suit their recommendation categories.

Instead of a numeric scale, we could have used a Likert scale of certainty, for example, the ‘Certainty of Response Index’, which ranges from ‘Totally Guessed Answer’ to ‘Certain’ [36]. However, we preferred not to use adjectives to measure uncertainty because of differences in how people interpret words like ‘likely’ [37]. We therefore used numeric responses that have less room for varying interpretation. A numerical scale may be more difficult to answer than one using qualitative descriptors; hence, we included an option for reviewers who could not answer the question. We also examined responses where the total percentage was not 100%.

We collected data on the reviewers’ characteristics that might influence their certainty, which were their gender, years working in research, and time spent on the review. Reviewers with more experience could be more certain as they become more confident. Reviewers spending more time could be more confident as they had potentially more thoroughly understood the paper. We collected gender because of previously documented differences in the peer review system by gender [38]. Reviewers were asked for their country, but this was only used to describe the sample and not to model uncertainty.

We collected data on the papers’ characteristics that might influence the reviewers’ certainty, which were the version, paper type, time between submission and review and readability. Later versions of the paper may have less uncertainty because the reviewers have had their initial questions answered. Paper type might be associated with certainty, as some papers may be less challenging for peer review. We tested whether protocols were different from other papers, as these papers could be less complex to review and there were a reasonable number of protocols in our sample (*Epidemiology* does not publish protocols and nor do many journals). In terms of review timing, slower reviews may be less certain, as delays might indicate the reviewer was unsure and needed to repeat their review, although delays could also be due to the reviewers being busy [39]. For readability, we examined the main text of the article and calculated the number of words and two readability scores: Flesch’s Reading Ease, which gives higher scores for shorter words and shorter sentences [40], and the New Dale–Chall, which measures comprehension difficulty [41]. Papers that are harder to read may have greater uncertainty. These predictors were only available for *F1000Research* where papers under review are openly available. A summary of the predictors is in electronic supplementary material, S3.

### 2.2. Statistical methods

We were interested in the overall levels of uncertainty. As a descriptive statistic, we calculated the number of reviews across all journals combined with no uncertainty, where the reviewer assigned 100% to one of the recommendations. We plotted the percentage of reviews with no uncertainty based on the reviewers’ recommendations.

We examined if the characteristics of the reviewer or the paper were associated with their percentages for the considered recommendations. We modelled the reviewers’ percentages using a Bayesian multinomial regression model using a probability scale (percentage ÷ 100). The multinomial model bounded the probabilities between 0 and 1 and allowed us to include predictors of the reviewers’ percentages and hence comment on changes in uncertainty. Our approach was a descriptive regression model not a predictive or causal model [42].

Each journal used different recommendation categories (e.g. ‘Accept’, ‘Approved’); hence, we used separate models for each journal.

**Figure 1.**
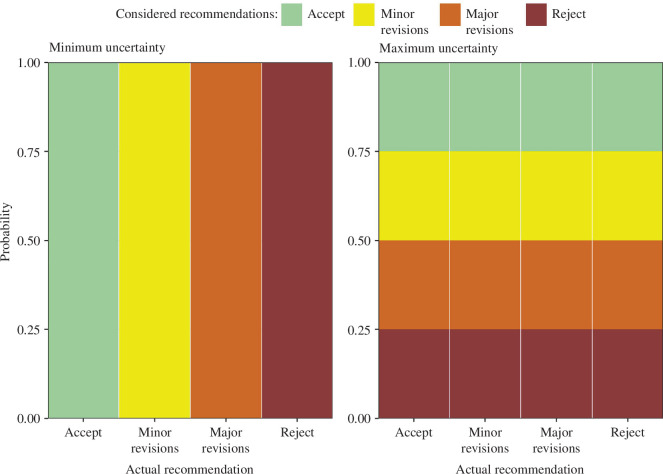
Illustrating how the reviewers’ percentages correspond to certainty or uncertainty using the four recommendations at *BMJ Open*. The plot shows the actual recommendation on the *x*-axis, and the bars are filled according to percentages of recommendations the reviewer considered. The left panel illustrates no uncertainty, as for each actual recommendation the reviewer only considered the same recommendation. The right panel illustrates maximum uncertainty as for each actual recommendation the reviewer gave equal consideration to every possible recommendation.

The multinomial model is illustrated in figure 1. We modelled the probabilities of each considered recommendation using the reviewers’ actual recommendation. For example, if a reviewer recommended ‘Accept’, then we modelled the probabilities they assigned to ‘Accept’ and all other recommendations.

We used the following three models. Detailed model equations are in electronic supplementary material, S4.

— Model 0: The considered probabilities only depended on the reviewer’s recommendation.
— Model 1: The considered probabilities depended on the reviewer’s recommendation and a predictor that was associated with the *mean* probabilities.
— Model 2: The considered probabilities depended on the reviewer’s recommendation and a predictor that was associated with the *uncertainty* in probabilities.

Model 0 is the baseline model, with which Models 1 and 2 are compared to assess whether the predictors are associated with the mean and/or uncertainty. We used 10-fold cross-validation to select the best model.

For Model 1, the predictor influenced the mean probability which was useful to answer questions such as whether female researchers had systematic differences in their probabilities.

For Model 2, an increase in the predictor increased uncertainty in all categories by moving the probabilities for all categories closer to 1/C , where C is the number of recommendation categories ( C=4 in figure 1). In visual terms, the predictor is modelled so that it can move the percentages away from the left panel of minimum uncertainty in figure 1 and towards the right panel of maximum uncertainty (or vice versa for an association in the reverse direction).

### 2.3. Missing data

We did not impute the reviewers’ percentages if they were missing, as this was the dependent variable [43]. We assumed missing predictors were missing at random and used multiple imputation using chained equations with 15 imputed data sets as 15% of observations had some missing data [44,45]. We used predictive mean matching to impute missing predictors, as the predictors were either categorical or had a skewed distribution. We compared the imputed results with those from a complete case analysis that excluded reviews with missing predictor data.

### 2.4. Protocol and sample size

We published a study protocol on the Open Science Framework [46]. In a change to our protocol, we included a model where the predictor impacted the mean (Model 1), as this provides a useful comparison to the models of uncertainty (Model 2). We planned to use the deviance information criterion as a measure of model fit, but found the estimated parameter numbers were not accurate and so we instead used 10-fold cross-validation to assess model fit.

We aimed to get at least 400 responses, as we believed this would provide sufficient data to investigate uncertainty over a range of paper and reviewer characteristics. Four hundred responses gave an 87% power to detect a 15% increase in uncertainty between two equally sized groups of 200. This estimate was calculated using simulated multinomial data with 400 simulated data sets [46]. This power calculation assumed all responses could be combined, but the three journals used different recommendation categories and so required separate analyses.

The study was approved by the Queensland University of Technology Human Research Ethics Committee (25 February 2022; LR 2022-5293-7535).

## 3. Results

**Figure 2.**
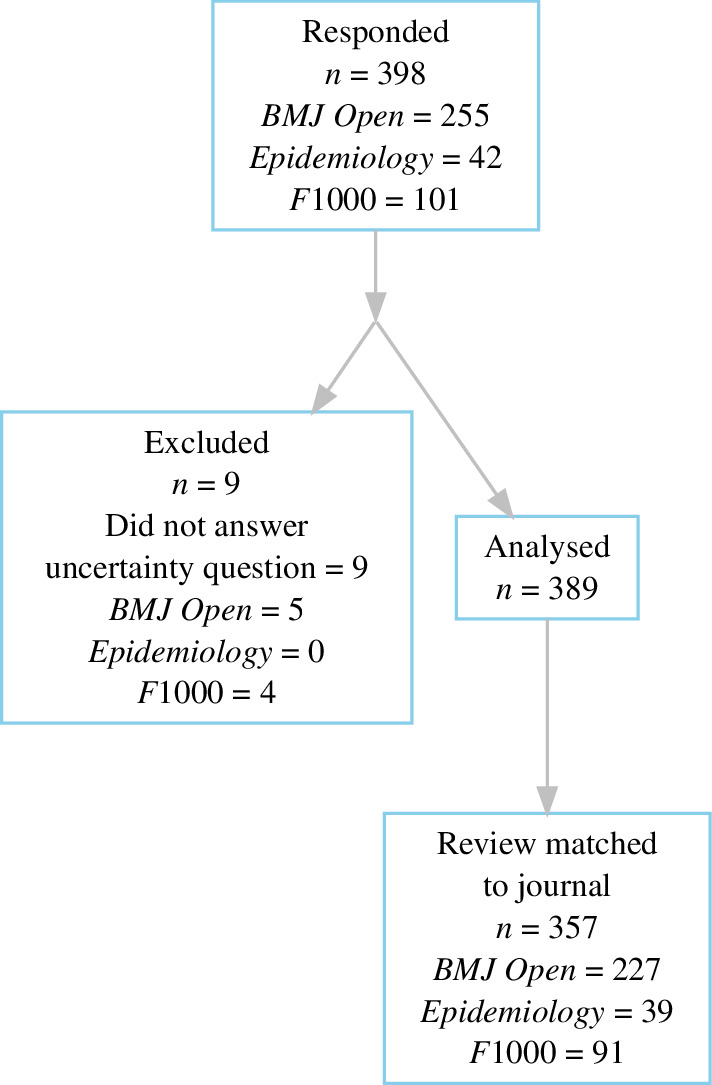
Flow chart of the number of responses and completed questions. The boxes show the overall numbers and the numbers per journal.

The number of responses is shown in figure 2. We were two reviews short of our target sample size of 400. Nine responses were excluded from all analyses, as they did not answer the uncertainty question. There were 32 responses that could not be matched to the journals’ records and so were excluded from any analysis that required journal information, for example, time to review. There were generally small levels of item-missing data; see electronic supplementary material, S5, for a graphical summary. The median time to complete the questions was 3 minutes.

The reviews were conducted between 29 March 2022 and 19 April 2023 (386 days).

The characteristics of the reviewers and papers they reviewed are summarized in table 1. The sample included a close-to-equal gender balance, but many reviewers were relatively established researchers, and only 12% had less than 5 years of experience. Most paper types were original research and were the first version. The median time taken to review was 3 hours, which is less than the median of 5 hours from an international survey [47].

**Table 1.** Characteristics of the reviewers and the papers they reviewed.

characteristic, summary statistics	category	statistics
journal, *n* (%)	*BMJ Open*	255 (64)
	*Epidemiology*	42 (11)
	*F1000Research*	101 (25)
reviewer’s gender, *n* (%)	female	177 (44)
	male	204 (51)
	non-binary/third gender	1 (<1)
	prefer not to say	5 (1)
	missing	11 (3)
reviewer’s years working in research, *n* (%)	less than 5 years	46 (12)
	6–10 years	108 (27)
	11–15 years	84 (21)
	16–20 years	45 (11)
	21 years or more	104 (26)
	missing	11 (3)
reviewer’s country (top five only), *n* (%)	USA	78 (20)
	Australia	46 (12)
	UK	36 (9)
	Canada	19 (5)
	Germany	14 (4)
time taken to review in hours, median (Q1–Q3)		3 (2–6)
paper type (top five only), *n* (%)	original research, original article	238 (60)
	protocol	76 (19)
	review, systematic review	12 (3)
	brief report	8 (2)
	software tool article	4 (1)
paper version, *n* (%)	1 (first submission)	304 (76)
	2 (first re-submission)	52 (13)
	3 (second re-submission)	6 (2)
	missing	36 (9)
time between submission and review in days, median (Q1–Q3)		56 (26–108)
number of words in the paper,^a^ median (Q1–Q3)		3246 (2450–4920)
Flesch readability,^a^ median (Q1–Q3)		28 (22–33)
Dale–Chall readability,^a^ median (Q1–Q3)		4 (1–9)

Q1 = first quartile, Q3 = third quartile.

^a^ Only available for *F1000Research*.

Eight reviewers (2%) said they could not answer the percentage question, but six still gave reasonable percentages and were included. There were 23 reviews (6%) where the percentage did not add to 100%. A plot of these percentages is in electronic supplementary material, S6.

### 3.1. Reviews with no uncertainty

**Figure 3.**
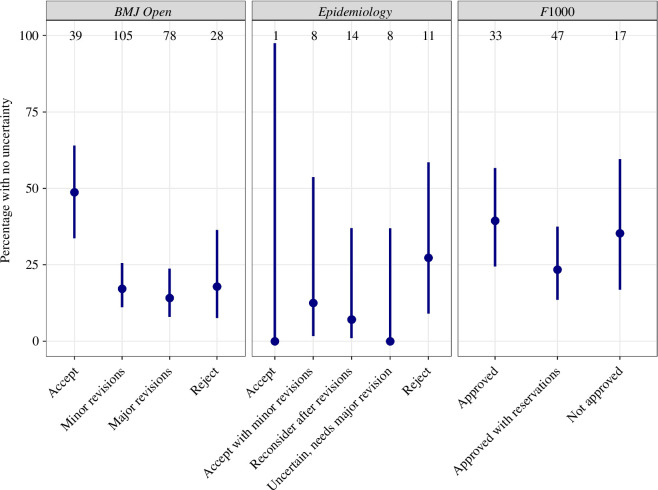
Percentage of reviews with no uncertainty by journal and actual recommendation. The estimates are the mean percentage (circle) and 95% confidence interval (vertical line) for the mean. The sample sizes are at the top of the panels.

Across the three journals, the percentage of reviews with no uncertainty was 23% with a 95% confidence interval from 19% to 27%. The percentage with no uncertainty by journal and recommendation is plotted in figure 3 and shows a generally V-shaped pattern with more certainty for the outermost recommendations. For *BMJ Open,* the percentage with no uncertainty was much greater for a recommendation of ‘Accept’ (49%, 95% CI 34–64%) than ‘Reject’ (18%, 95% CI 8–36%).

### 3.2. Average percentages

**Figure 4.**
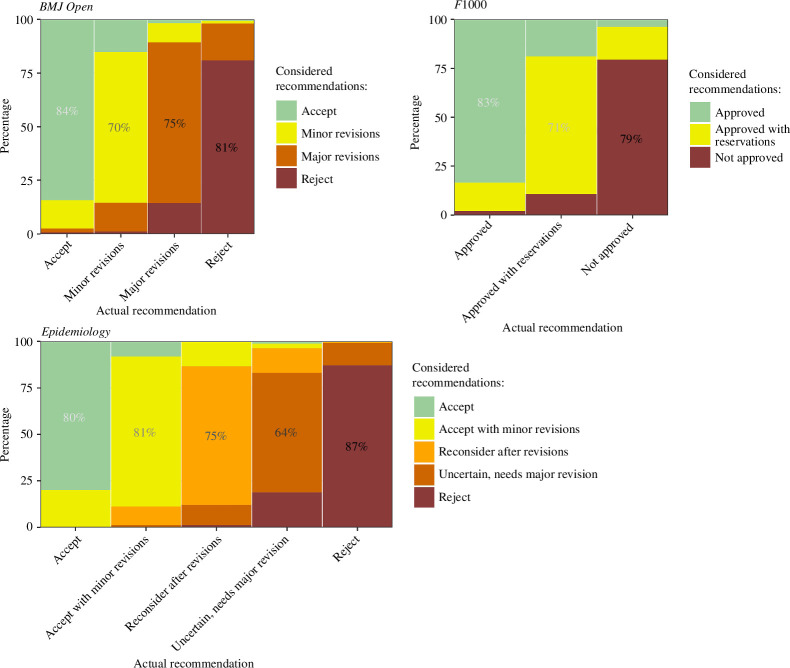
Average probabilities of each considered recommendation using the reviewers’ actual recommendations. Results are presented by journal. The modal percentage is added for each bar.

The average considered percentages by journal and the reviewers’ actual recommendation are in figure 4. For each recommendation, the modal percentage is that same recommendation, with generally larger percentages (greater certainty) for the outermost recommendations. The lowest mode is 64% for the recommendation at *Epidemiology* of ‘Uncertain, needs major revision’.

The other recommendations considered were most often neighbouring recommendations to the actual recommendation. There were only small percentages for the opposite recommendation. For example, at *BMJ Open* for reviewers that recommended ‘Accept’ the average percentage considering ‘Reject’ was under 1%, and similarly for reviewers that recommended ‘Reject’ the average percentage considering ‘Accept’ was also under 1%.

### 3.3. Models of the reviewers’ percentages

Only two predictors were associated with the reviewers’ percentages, and these were the reviewer’s gender and whether the paper was a protocol. The model fit results are summarized in electronic supplementary material, S7.

**Figure 5.**
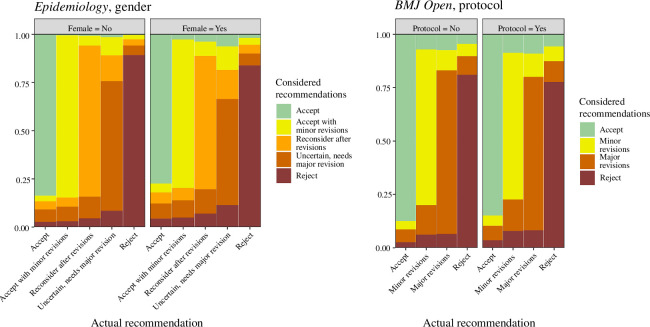
Estimated reviewer probabilities for the considered recommendations by actual recommendation. The results are the fitted probabilities using Model 2.

Gender was important at *Epidemiology,* as female reviewers were associated with more uncertainty, according to Model 2. When female reviewers recommended ‘Accept’, they gave more consideration to other recommendations with a lower probability of ‘Accept’, mean difference compared with other reviewers: 0.06 (95% CI: 0.04–0.08). This difference can be seen in the ‘Accept’ bars in the fitted values in figure 5 (left panel). Similarly, if female reviewers recommended ‘Reject’, they gave a lower probability to ‘Reject’, mean difference compared with other papers 0.05 (95% CI: 0.04–0.07). The fitted values are shown using the imputed data in electronic supplementary material, S8, and show little difference between the models using the complete case data or imputed data.

Protocol papers were associated with more uncertainty at *BMJ Open*, according to Model 2. For reviewers examining protocol papers who recommended ‘Accept’, they gave a lower probability to ‘Accept’ mean difference: 0.03 (95% CI: 0.02–0.04).

## 4. Discussion

In this first study of uncertainty among reviewers, we found that most reviewers reported some uncertainty in their recommendations, but the percentages given to other recommendations were generally small (figure 4). Uncertainty was smallest (on average) for the outermost recommendations and greatest for the central recommendations (figure 3). High- and low-quality papers may be ‘easy to detect’ [48], with the central recommendations in a grey zone where reviewers are undecided—one central recommendation wording specifically includes uncertainty: ‘Uncertain, needs major revision’ at *Epidemiology*. However, it is also possible for a reviewer to be certain that they need more information to judge a paper, so reviewers could have assigned 100% to the central recommendations.

Within-reviewer uncertainty has likely impacted reviewers’ recommendations and editorial decisions and hence impacted researchers’ careers. It is part of the ‘luck of the draw’ in peer review [49]. A recent recommendation is to embrace this uncertainty and randomize decisions in the ‘grey zone’ [50], and similarly, the editor of a prestigious journal joked about the randomness of their decisions [3]. If a journal was bold enough to use partial randomization in its decisions, then the reviewers’ uncertainty could be used to select which submissions are randomized. Another large change to standard peer review occurred at the journal *eLife* where papers are no longer subjected to binary reject/accept decisions and reviewers are not asked to make recommendations [51]. The motivation for this change was to capture some of the nuance and ambiguity in peer review.

There was more uncertainty for ‘Reject’ than ‘Accept’ recommendations at *BMJ Open* (figure 3). A review of studies of between-reviewer reliability found a generally higher agreement for ‘Reject’ than ‘Accept’ recommendations, the reverse of our results within reviewers [52]. Reviewers at journals with open peer review (*F1000Research* and *BMJ Open*) may prefer to be more certain about papers they accept because their reputation is partly on the line once the paper is published.

We found more uncertainty for female reviewers at *Epidemiology*. A review of studies that examined a self-confidence gap by gender found that experts in the field thought that men were much more self-confident than women but a meta-analysis showed only a small difference [53]. The gender difference we found was small and only in one journal. The small increase in uncertainty for female reviewers may be a positive attribute, as it may show greater humility and a more considered recommendation [54].

The greater uncertainty we found for protocols may be because it is harder to judge planned research compared with completed research. A similar result was found in a between-reviewer reliability study of funding peer review, where fellowship applications had a higher reliability in funding decisions compared with project applications, possibly because fellowships mostly concern the researchers’ past work, whereas projects are largely concerned with planned research [55]. However, the absolute difference in probability that we found between protocols and other papers was small (figure 5). One reviewer commented on reviewing a protocol: ‘I always find these very difficult to review as I am somewhat unclear as to the real purpose of peer-review here’. So the increased uncertainty when reviewing protocols could be due to a lack of experience reviewing protocols compared with standard papers and/or a lack of guidance on how to review protocols. Reviewers may be unsure if they are assessing the quality of the protocol or the quality of the reporting. Journals may need to provide additional guidelines for reviewers considering protocols.

Some uncertainty may have occurred because a reviewer only felt qualified to review parts of a paper, e.g. lacking the competence to judge the statistical methods [56]. One study participant commented, ‘Was outside my main area of expertise, which contributed to my uncertainty’. One suggestion is to segment papers so peer reviewers can comment on those sections where they are qualified and hence more certain [57]. Ethical reviews of proposed research are already somewhat segmented, as review boards often include expertise from lawyers and clinicians [58]. Segmented review at journals may be worth experimenting with, and our uncertainty question could be used to compare the reviewers’ certainty between a standard and segmented peer review system. Another way to partially segment reviews is to use automated reviews to examine compliance with journal policies and reporting quality, potentially giving human reviewers more time to focus on difficult concepts such as novelty and impact [59]. As the number of published papers increases [60], the burden on peer reviewers will also increase [61], and hence more studies on peer review processes are badly needed.

One potential cause of uncertainty is whether the paper is well written, and one participant commented on this, ‘This paper needs a lot of work to be understandable […] many of the problems are related to use of English’. However, we did not find any association between uncertainty and readability based on the length of the paper and two readability scores, although we could only examine this in one journal and the predictors we used may not accurately measure scientific readability. Some authors are turning to large language models to help them write papers and grants [62,63], which could increase their readability, but with reasonable concerns around paper mills and artificial intelligence ‘hallucinations’ [64].

### 4.1. Potential actions for publishers

Our uncertainty question could be used by journals to improve their peer review processes. For example, if a paper receives two reviews where both reviewers express a large uncertainty (with the threshold determined by the journal), then a third review may be sought. Some journal editors already use this approach when two reviews have conflicting recommendations [65,66].

Our uncertainty question could be a key study outcome when journals experiment with their peer review systems [32]. For example, a randomized trial of reviewer training or a pre–post study of a change in how reviewers are selected. If an intervention reduced reviewer uncertainty, then it might be preferred even if, for example, it was also associated with an increase in ‘Reject’ recommendations.

We only examined three journals, but we think it is likely that within-reviewer uncertainty will occur at other journals. We encourage all journals to consider this uncertainty and the implications for what papers they publish. Journals could ask the uncertainty question for randomly selected reviews (possibly just 1% to 2%) which would allow them to track uncertainty over time and look for patterns in uncertainty, e.g. greater uncertainty for papers from non-native English-speaking countries. However, some reviewers may be unwilling to admit uncertainty directly to the journal for fear of appearing incompetent.

There is evidence that the provision of systematic training for researchers on how to provide useful and constructive peer review remains lacking. A survey of peer reviewers in nursing found that 65% wanted training and 87% wanted feedback from editors about their review [67]. In a large international survey of peer reviewers, 68% thought that formal training would improve the quality of reviews [68]. However, editors seldom ask peer reviewers if they have been trained before inviting them to review for a journal, and where peer review training is available, researchers are not required to participate and uptake of training can be low. Given that peer review is a core research competency, it is important to ensure that the provision of training is both available and encouraged. In an ideal world, researchers would be motivated to provide peer review in ways that contribute to their career development and enhance the research culture more broadly.

### 4.2. Future work

Applying our uncertainty question to funding peer review would be interesting, as these decisions can be the most crucial for researchers’ careers. Reviewers for funding applications often review multiple applications, and this would be statistically useful for separating the variance in uncertainty due to the characteristics of the application and the reviewer. Some funding systems ask reviewers to submit scores instead of a categorical recommendation, and the uncertainty in scores could be captured by asking reviewers for the range of scores that they considered.

Another important source of uncertainty is the editor’s decision. These decisions can be especially difficult when reviewers have conflicting opinions about a paper’s merits. We did not examine editor uncertainty, but editors could similarly be asked about their uncertainty using the numerical estimate of uncertainty.

### 4.3. Limitations

Our question on reviewer uncertainty is new and has not been validated. We have relied on its face validity, but we do not have data on its validity and reliability. Two authors have experience in economic modelling, where uncertainty is sometimes estimated by asking experts for the lower and upper ranges in their estimates (e.g. estimating the cost of a day in hospital [69]). There is a long history of eliciting probability distributions from experts to use as priors in Bayesian models [35].

The three journals each had their own recommendation categories, and therefore it was not possible to combine the results. Splitting the data reduced the statistical power to find associations between uncertainty and the predictors, although we were able to detect relatively small differences in probabilities for reviewer gender and protocol papers. We observed differences between the three journals in terms of what predictors were associated with uncertainty, and we should therefore be cautious about generalizing the results to other journals.

We do not have data on how many reviewers were invited to participate and hence could not calculate a response rate. There could be selection bias in those taking part in our study, as participants may be more likely than the wider population of reviewers to be interested in uncertainty and perhaps more likely to admit uncertainty. We tried to reduce response bias by using a short questionnaire, and the median time to complete the questions was just 3 minutes.

We may have missed characteristics about the paper and the reviewers that could have explained some of the variance in uncertainty. For example, if the paper was based on funded research that has already been through peer review, then reviewers who like the paper may be more confident in recommending acceptance. Characteristics concerning the study design may also be associated with reviewer uncertainty, including the sample size and whether the study was a randomized trial. A qualitative study of peer reviewers’ uncertainty would be helpful.

We may have received multiple responses from the same reviewer, but we did not collect personal data on reviewers and were unable to adjust for this correlation. Given the size of the reviewer pool [70], we believe any non-independence will be small.

We did not examine the reviewers’ comments to examine a correlation between their comments and their uncertainty.

## Data Availability

The data and R code are openly available on GitHub [71] and Zenodo [72]. Supplementary material is available online [73].
